# Decoding the evidence: A synopsis of indications and evidence for catheter ablation in atrial fibrillation (Review)

**DOI:** 10.3892/mi.2024.200

**Published:** 2024-11-05

**Authors:** Omar Obeidat, Mohamed F. Ismail, Saeed Abughazaleh, Hashim Al-Ani, Mohammad Tarawneh, Laith Alhuneafat, Ali Obeidat, Abedallah Obeidat, Qusai Alqudah, Moh'd Alamin Daise, Hamza Alzghoul, Mohammad Al-Hammouri, Ward Althunibat, Ann Tong, Mazahir Alimohamed

**Affiliations:** 1 Graduate Medical Education Program, College of Medicine, University of Central Florida, Orlando, FL 32827, USA; 2 Internal Medicine Residency Program, HCA Florida North Florida Hospital, Gainesville, FL 32605, USA; 3 St. Elizabeth's Medical Center, Boston University Teaching Hospital, Brighton, MA 02135, USA; 4 Division of Cardiovascular Medicine, University of Minnesota, Minneapolis, MN 55455, USA; 5 Division of Cardiology, Department of Internal Medicine, Faculty of Medicine, Jordan University of Science and Technology, Irbid 22110, Jordan; 6 Internal Medicine Residency Program, HCA Florida Ocala Hospital, Ocala, FL 34471, USA; 7 Department of Cardiology, The Cardiac and Vascular Institute, Gainesville, FL 32605, USA; 8 Department of Cardiology, Essentia Health, Duluth, MN 55805, USA

**Keywords:** atrial, fibrillation, catheter, ablation

## Abstract

The present study reviews the role of catheter ablation (CA) in the management of atrial fibrillation (AF), a widespread arrhythmia associated with increased morbidity and mortality. The present review explores current indications and recent evidence supporting CA, assessing patient outcomes and identifying common complications associated with the procedure. Emphasis is placed on optimizing risk factors prior to ablation, including weight control and hypertension management, as these measures can significantly enhance post-procedural outcomes. The present review also discusses the use of antiarrhythmic and anticoagulant therapies following CA to minimize recurrence and reduce stroke risk. Additionally, the cost-effectiveness of CA is discussed, comparing its long-term economic impact with that of medical therapy alone. The present comprehensive review provides insight into best practices for AF management, supporting CA as a promising approach when integrated with targeted lifestyle modifications and pharmacological support for improved, patient-centered outcomes.

## 1. Introduction

Atrial fibrillation (AF) is a common cardiac arrhythmia that affects millions of individuals worldwide. The prevalence of AF has exhibited a 3-fold increase over the past 50 years according to data from the Framingham Heart Study and the American Heart Association (AHA) (1,2). It is characterized by an irregular and often rapid heartbeat, which can lead to several serious complications, such as stroke, heart failure and a decreased quality of life (3). The condition is caused by abnormal electrical signals in the heart that disrupt the normal rhythm of the atria (4). Several risk factors have been linked to the development of AF, many of which are modifiable and include hypertension, obesity, obstructive sleep apnea (OSA) and alcohol consumption (4).

The primary goals of treating patients with AF include managing symptoms, controlling the heart rate or rhythm, and reducing the risk of stroke (5). Catheter ablation (CA) is an established treatment method for improving symptoms, rate or rhythm control and is the most common procedure performed in electrophysiology. Evidence from randomized controlled trials (RCTs) also supports the effectiveness of CA in patients with comorbid AF and heart failure (5-8). The present review discusses the current and evolving indications for CA in patients with AF, summarizing the safety and efficacy of CA procedures, and reviewing the data that support the significance of lifestyle modifications for improving outcomes after ablation.

## 2. Current indications and evidence in the literature

CA, a highly sophisticated and complex procedure, requires a thorough and rigorous assessment of its potential risks and benefits prior to its implementation as a therapeutic strategy in AF (9). To ensure optimal outcomes, it is imperative that the procedure be executed by highly qualified and experienced electrophysiologists or surgeons operating in specialized centers. Furthermore, the indications for CA should be determined by the specific subtype of AF of the patient's and the previous response of the patient to class I or III antiarrhythmic medications, as established by the current literature. These considerations are of paramount importance in ensuring the safe and effective utilization of CA as a therapeutic option for AF and are vital for achieving successful long-term outcomes for patients with AF (1-9).

In addition to these considerations, patient characteristics such as the presence of comorbid conditions such as heart disease, obesity, and sleep apnea, as well as factors such as left atrial size, age, frailty, and duration of continuous AF, may predict a lower success rate or higher complication rate in the management of AF (9). Therefore, it is essential to take these variables into account when managing and treating patients with AF to ensure the best possible outcomes. Table I summarizes the indications with the level of evidence.

### CA as a first-line therapeutic option

First-line therapy with AF ablation, prior to the administration of class I or class III antiarrhythmic agents, has been extensively evaluated in the literature concerning both symptomatic paroxysmal and persistent AF (9-22). This approach is linked to a considerable decrease in the recurrence of arrhythmias, significant enhancements in symptoms related to arrhythmia, and improved quality of life. Moreover, ablation is associated with a reduced frequency of adverse events. Additionally, CA is associated with markedly lower rates of disease progression, indicating its role as a disease-modifying intervention (23).

A recent meta-analysis conducted by Turagam *et al* (24), which included six randomized controlled trials and a total of 1,212 participants, demonstrated that the ablation group exhibited a significantly lower recurrent atrial arrhythmia rate [risk ratio (RR), 0.61; 95% CI, 0.51-0.74], and a reduced incidence of symptoms and hospitalization (RR, 0.44; 95% CI, 0.27-0.72 and RR, 0.32; 95% CI, 0.19-0.53, respectively). Additionally, there was no statistically significant difference in the incidence of adverse events between the two groups (RR, 1.52; 95% CI, 0.81-2.85). The most commonly observed adverse effect in the ablation group was cardiac effusion, while bradycardia was observed in the medication group.

These findings are consistent with the 2016 European Society of Cardiology (ESC) guidelines for the management of AF and the 2014 American College of Cardiology (ACC)/AHA/Heart Rhythm Society (HRS) guidelines for the management of AF. A consensus statement on AF ablation in 2017 established that the level of evidence for utilizing ablation as a primary line of treatment without a trial of antiarrhythmic agents is class IIa indication in both paroxysmal and persistent symptomatic AF, and class IIb in long-standing symptomatic AF (9-11). In the 2023 ACC/AHA/ACCP/HRS guidelines CA is recommended as first-line treatment in selected symptomatic patients with paroxysmal AF who is generally young with fewer comorbidities to improve symptoms and reduce progression to persistent AF (class 1a recommendation) (25).

In patients with symptomatic pauses (tachy-brady syndrome), CA is considered the preferred treatment option. In these cases, initiation of medical therapy in the absence of a permanent pacemaker has been demonstrated to increase morbidity and mortality. Several studies have reported that CA is an effective treatment option, resulting in the resolution of symptoms without the need for a pacemaker (26-28).

Furthermore, CA is recommended as a primary line of treatment in highly competitive athletes as several studies have demonstrated favorable outcomes in this patient population (29-31).

### CA as second-line treatment

In the 2017 consensus and 2023 ACC/AHA/ACCP/HRS guidelines, CA is indicated as a treatment option for patients who have failed one or more antiarrhythmic medications, did not tolerate the medications or the antiarrhythmic medication is not preferred and continued rhythm control is desired and classified as a class IA recommendation (9,25). This classification is based on a significant body of clinical research, with >16 randomized trials having been conducted to evaluate the effectiveness of CA in this population (9,25,31). A recent meta-analysis by Deshpande *et al* (32) in 2022 included a total of 4,822 patients and found that the risk of arrhythmia recurrence was significantly lower in the ablation group, with an odds ratio of 0.25 (95% CI, 0.18-0.36). Additionally, all-cause mortality was also found to be significantly lower in the ablation group, with an odds ratio of 0.33 (95% CI, 0.17-0.63). There were no significant differences between the two groups in terms of stroke/transient ischemic attacks (TIAs), bleeding and cardiovascular mortality.

### CA in patients with heart failure and reduced ejection fraction

The loss of atrial contraction in AF impairs left ventricular filling, potentially reducing cardiac output by up to 25%. The irregular and rapid conduction of impulses in AF may lead to left ventricular dysfunction (33,34). The restoration of sinus rhythm can improve stroke volume and may reverse cardiac remodeling. This may explain the rapid improvements in hemodynamics observed in patients following the re-establishment of sinus rhythm, and it also clarifies why some patients with heart failure exhibit rapid hemodynamic improvements after returning to sinus rhythm (33-35).

In the 2019 2019 AHA/ACC/HRS Focused Update guidelines, CA as a treatment option for heart failure patients was classified as a class IIb recommendation (18). This classification is based on a significant body of clinical research, including numerous clinical trials and meta-analyses, which have evaluated the safety and outcomes of this treatment in this patient population (9,36-38). A recent meta-analysis conducted by Şaylık *et al* (37) in 2022, which included a total of 2,187 patients, demonstrated that CA was associated with a lower risk of all-cause mortality with a relative risk of 0.64 (CI: 0.5, 0.82). Additionally, this study found that patients who underwent CA had greater improvement in left ventricular ejection fraction (LVEF) with a mean difference of 5.38 (CI: 1.80, 8.97), as well as an improved quality of life, as demonstrated by a greater reduction in scores on the Minnesota Living with Heart Failure Questionnaire [MD=-9.59 (CI: -16.72, -2.45), P<0.01], and longer 6-min walking distances compared to patients in the medical therapy group [MD=20.3; (CI: -4.37, 44.9)]. Furthermore, a meta-analysis by Chang *et al* (38) demonstrated similar findings and reported lower rates of heart failure hospitalization and AF recurrence among patients who underwent CA.

### CA in patients with heart failure with preserved ejection fraction (HFpEF)

AF is commonly observed in patients diagnosed with HFpEF, characterized by an LVEF of 50% or greater. The prevalence of AF in this patient population ranges from approximately 40 to 60%, and both conditions are typically associated with advancing age (39,40).

Patients with HFpEF who also have AF exhibit a poorer prognosis compared to those in sinus rhythm. The hazard ratio for combined all-cause mortality or heart failure hospitalizations in patients with HFpEF with AF was 1.365 (95% CI, 1.152-1.619; P<0.001) (39).

A recent meta-analysis conducted by Gu *et al* (41) evaluated the effectiveness of CA in the treatment of HFpEF and without heart failure. Their study included a total of 1,696 patients and found that CA was effective in maintaining sinus rhythm in the HFpEF group and was non-inferior to those without heart failure with a relative risk of 0.92 (95% CI, 0.76-1.10; P=0.34) (41). Additionally, their study found that CA significantly improved the maintenance of sinus rhythm with a relative risk of 4.73 (95% CI, 1.86-12.03; P=0.001) and reduced rehospitalization for heart failure compared with medical therapy with a relative risk of 0.36 (95% CI, 0.19-0.71; P=0.003). However, their study found no significant differences between the two groups in terms of mortality rate (P=.59). Overall, that study provides evidence supporting the effectiveness of CA as a treatment option for patients with HFpEF and without heart failure (41). Other studies also support these findings (9,42).

### CA in the elderly

AF is a common condition among older adults (≥65 years), and there have been numerous studies that have specifically focused on evaluating the outcomes of AF ablation in this population. However, the safety and efficacy of CA as a treatment for AF in older individuals is a topic of ongoing debate in the medical community. Meta-analyses have yielded conflicting results, with some studies suggesting non-inferiority in terms of recurrence rate compared to younger populations, while others have reported inferior efficacy. Despite these inconsistencies, there is a consensus among medical experts that the complication rate, including cerebrovascular accidents, bleeding, and mortality, is higher in older patients who undergo CA (9,41-46).

In view of these findings, the 2017 consensus and 2023 ACC/AHA/ACCP/HRS guidelines recommend that CA be considered as a treatment option for selected older individuals with AF, with similar indications as for younger patients (class IIa recommendation) (9,25). However, it is should be noted that older patients may have a higher need for concomitant antiarrhythmic medication after CA compared to younger patients (46).

### CA in asymptomatic patients

The prevalence of asymptomatic AF in the literature ranges from 10 to 40%, with higher prevalence in males and in older age groups (47). Some studies have reported conflicting data regarding the cardiovascular risk and stroke in asymptomatic patients, with some studies reporting less cardiovascular disease and a lower long-term risk overall in comparison to symptomatic patients (47,48), while other studies have reported higher or similar risk to symptomatic patients (47,49,50).

Several studies have compared the results of CA in asymptomatic patients to those of symptomatic patients. The study by Mohanty *et al* (51) included 61 asymptomatic patients with longstanding persistent AF and reported that 57% of the patients maintained sinus rhythm at 20 months of follow-up, with significant improvement in quality of life and exercise capacity. In total, 25 patients had a recurrence of AF after ablation; among these, 21 patients (34% of the total number of patients included) were symptomatic after the recurrence. The study by Wu *et al* (52) included 66 asymptomatic patients with asymptomatic persistent AF, and 35% of the patients maintained sinus rhythm at 1-year follow-up. Symptoms scores improved significantly across 6 of 8 measures, suggesting that the patients were not truly asymptomatic. 43 (65.15%) patients had a recurrence; among these, 16 patients (24% of all patients) became symptomatic.

The study by Pak *et al* (53) included a total of 5,013 patients from the Kansai Plus Atrial Fibrillation (KPAF) Registry, 64.4% of the patients had paroxysmal AF, 22.7% had persistent AF and 13% had long-standing AF. They reported no significant difference between symptomatic and asymptomatic patients as regards the recurrence of the supraventricular arrhythmia in 4 years of follow-up; 37.5 vs. 40.6% (P=0.6) in paroxysmal AF, 45.2 vs. 55.1% (P=0.09) in persistent AF and 59.3 vs. 63.6% (P=1.0) in long-standing AF. In addition, there was no significant difference between the two groups in cardiovascular, cerebral and gastrointestinal events during the 4 years of follow-up following CA; 7.1 vs. 6.8% (P=0.7) in paroxysmal AF, 5.4 vs. 8.7% (P=0.3) in persistent AF and 4.4 vs. 5.1% (P=0.5) in long-standing AF (53).

Another study by Kawaji *et al* (54) compared CA to conservative management in patients with symptomatic and asymptomatic AF. They included 537 AF patients, and the median follow-up period was 5.3 years. The CA group was associated with a significantly lower incidence of composite cardiovascular death, heart failure hospitalization, ischemic stroke, or major bleeding, (14.7 vs. 25.4% at 8 years; log-rank P=0.008). However, this advantage was significant only in patients who had previous AF-related complications (19.2 vs. 55.6% at 8 years; log-rank P=0.006), but not among those without any complication (13.9 and 17.3%; P=0.08). On the other hand, among symptomatic patients, the benefit was regardless of previous AF-related complications (54).

The 2017 consensus and 2023 ACC/AHA/ACCP/HRS guidelines recommend that AF ablation can be considered in selected (younger patients with minimal comorbidities and a moderate to high burden of AF or persistent AF) patients with symptomatic or minimally symptomatic AF as a class IIb recommendation. The procedure required discussion with the patient regarding risks, and benefits (9,25). However, it should be noted that the benefit of the procedure in asymptomatic patients may be uncertain, and further research is required to establish the optimal course of action for these patients.

### CA to reduce stroke risk

Patients undergoing CA often hope to avoid long-term oral anticoagulation therapy. A sub-study from the AFFIRM trial (55) reported that maintenance of sinus rhythm and oral anticoagulation are associated with an improved survival and a 60% reduction in the risk of stroke. Hence, it is inferred that CA, with its higher rates of patients maintained in sinus rhythm, would be associated with a reduced risk of stroke. However, CA was not used as a strategy for rhythm control in the AFFIRM trial. Clinical trials and meta-analyses from randomized controlled trials do not support this theory (9,32,56-59). To date, 20 clinical trials have compared CA to medical therapy. The overall risk of stroke was low in both the CA group (0.85) and the medical therapy group (57). The incidence of stroke did not differ significantly between the two groups in all randomized control trials. Meta-analyses of clinical trials, such as those by Deshpande *et al* (32), Barra *et al* (56), Mao *et al* (58) and Shi *et al* (59), all reported no difference in stroke incidence between the two groups. However, the study by Barra *et al* (56) included a separate analysis of cohort studies and registries, in which a significant difference was found, with CA being associated with a reduced risk of stroke and cerebrovascular accident (2.3 vs. 5.5%; RR, 0.57; 95% CI, 0.46-0.70; P<0.001; I^2^=62%) (56).

One possible explanation for the lack of significant impact of CA on the risk of stroke in randomized controlled trials is the low incidence of stroke and low baseline risk in these trials. Out of 44 strokes reported in all trials, 29 were reported in two trials [CASTLE-AF (8) and CABANA trials (6)], with the remaining trials reporting only 15 stroke cases (32,56-59). The annual incidence of stroke among patients undergoing CA is typically low, regardless of the outcomes of the procedure, due to the inherently low baseline risk. The study by Freedman *et al* (60) reported that the risk of stroke in patients with AF who were on anticoagulation therapy was similar to that of patients without AF and was primarily related to patient risk factors, such as age, sex and comorbidities, rather than anticoagulation failure.

Therefore, it would be challenging to demonstrate a significant additional benefit of CA in reducing residual stroke risk beyond that of proper anticoagulation. Even if ablation were to lower the risk of stroke by 50%, the number of patients required to undergo the procedure to prevent one stroke event would still be high, given the low baseline risk of 1.0% in patients receiving medical treatment, as seen in randomized studies. This is why no randomized study has yet been able to demonstrate a meaningful impact of AF ablation on stroke rates, and why even combined data lacks sufficient statistical power. The low incidence of stroke in randomized trials is indeed reassuring, although one must exercise caution when generalizing these results to real-world patients. Although outcomes in randomized trials may not always reflect real-world patient populations, some studies have shown that the incidence of stroke among real-world patients aligns with the rates reported in landmark trials such as ROCKET-AF and RE-LY (61,62).

## 3. Outcomes of the procedure

### Success rate

The success rates of percutaneous AF CA vary considerably, ranging from 50 to 80% (9-47). However, accurately estimating the success of CA for AF is challenging due to inconsistencies in the definitions of procedural success and post-procedural recurrences, variations in the intensity of post-procedural rhythm monitoring, and differences in outcome analyses after single or multiple AF ablation procedures (9,11,25).

The academic community has established a definition for AF recurrence, which refers to the occurrence of any symptomatic or asymptomatic atrial tachyarrhythmia lasting >30 sec after the AF-CA procedure (9,11,25). However, it is important to recognize that this definition should be understood within the broader clinical context, which includes factors, such as the improvement of symptoms, the reduction of heart failure symptoms and improvements in LVEF, and overall quality of life. These broader clinical outcomes should be considered when assessing the success of AF-CA, beyond the strict definition of AF recurrence alone. A summary of the outcomes of the procedure is presented in Table II.

### Success rate after single and multiple procedures

There is increasing evidence from the literature to suggest that the long-term success rates for AF ablation are significantly improved following repeated procedures compared to a single procedure (63-65). A previous meta-analysis of 19 studies involving patients with 6,167 AF with an average age ranging from 51 to 65 years demonstrated that repeated AF ablation procedures were associated with significantly improved long-term success rates compared to a single procedure (63). The results of that study revealed that following a single CA procedure, the rate of freedom from AF was 65% at 1 year, 56% at 3 years, and 51% at 5 years. However, following multiple ablation procedures, AF was successfully suppressed in 86% of patients at 12 months, 79% of patients at 3 years, and 78% at 5 years (63).

Moreover, studies have shown that the success rate for a single-procedure CA is higher in patients with paroxysmal AF than in those with persistent AF (63-65). It was found that following 3-5 years of follow-up, only 54% of patients with paroxysmal AF and 42% with persistent AF were found to be arrhythmia-free (63). Nevertheless, following multiple procedures, long-term success rates were similar in patients with paroxysmal and persistent AF (79 and 78%, respectively), although the number of procedures per patient was significantly higher in patients with persistent AF than in those with paroxysmal arrhythmia (63). These findings highlight the potential benefits of repeated ablation procedures for achieving better long-term outcomes in AF patients, especially those with persistent AF.

### Quality of life

The primary goal of treating patients with AF is to improve their symptoms. Thus, making formal evaluations of quality of life is important in determining the success of ablation procedures (66,67). These evaluations offer a more comprehensive view of symptom change, arrhythmia burden, health and function, compared to only monitoring rhythm status. For this purpose, various tools are used, such as the SF-36 health survey, which can be utilized for a wide range of health conditions (68), and AF-specific questionnaires (67,69). However, it should be noted that both types of quality of life instruments depend on the subjective experience of the patient and there is no agreement on which is superior. Consequently, utilizing a standard instrument, such as the SF-36 in the assessment of AF can still yield valuable insight into the quality of life of an individual.

Numerous studies have demonstrated that CA can significantly improve quality of life in patients with AF compared to antiarrhythmic drug (AAD) therapy (67,70-75). Non-randomized studies have consistently shown sustained improvements in quality of life scores following 12 months of AF ablation (76), while in randomized clinical trials comparing ablation as first-line therapy, quality of life improved to a greater extent with ablation than with AAD treatment (72-76).

However, the duration of improvement in quality of life following CA vs. AAD therapy remains a topic of discussion. A previous meta-analysis of 12 randomized controlled trials involving 1,707 patients with symptomatic AF, which compared CA as first or second-line therapy with AAD treatment, found that at a 3-month follow-up interval, CA led to greater improvements in various aspects of the SF-36 questionnaire and in the symptom frequency score compared to AAD treatment (77). Although the differences between the two treatments decreased over time, beyond 9 months, there were no significant differences in any of the quality of life metrics, symptom frequency, or severity scores (77).

Notably, patients who remain AF-free following ablation exhibit greater improvements in quality of life than those with recurrent arrhythmia (70-77). However, even patients with AF recurrence exhibit significant improvements in quality of life following ablation compared to pre-ablation values, possibly due to a higher proportion of asymptomatic arrhythmia episodes, reduction in AF burden, left atrial denervation, increased AAD effectiveness, or a placebo effect post-procedure (78). These findings highlight the complexity of the association between AF ablation and quality of life outcomes, and suggest the need for the careful consideration of patient-specific factors when discussing the potential benefits and risks of ablation.

### Mortality

Randomized trials and metanalyses of the trials have not demonstrated the superiority of ablation compared to medical therapy in reducing mortality, with the exception of patients with heart failure with reduced ejection fraction as aforementioned, most probably due to the limited duration of post-procedural follow-up and the selection of relatively younger patients with a low prevalence of structural heart disease and low thromboembolic risk (9,22,35-38,79). However, several non-randomized studies have demonstrated the advantages of CA over medical therapy with respect to survival in the subgroups of ‘sicker’ and older patients with AF with significant heart disease, HF, and a high CHA2DS2VASc score (80-85). These studies included patients with a mean age between 57 and 69 years and more than one risk factor for thromboembolism; more than half of the patients had significant structural heart or lung disease (80-85). During the mean follow-up of 2.5-4.4 years, a significantly lower mortality rate (3-6 vs. 7-14%) was observed following ablation compared to AAD therapy. Moreover, a stable sinus rhythm after the procedure was strongly associated with a reduction in mortality [hazard ratio (HR), 0.14; 95% CI, 0.06 to 12.36], while an AF recurrence was an independent predictor of mortality (HR, 2.52; 95% CI, 1.05 to 6.06) (81,83).

## 4. Complications

**Figure 1:**
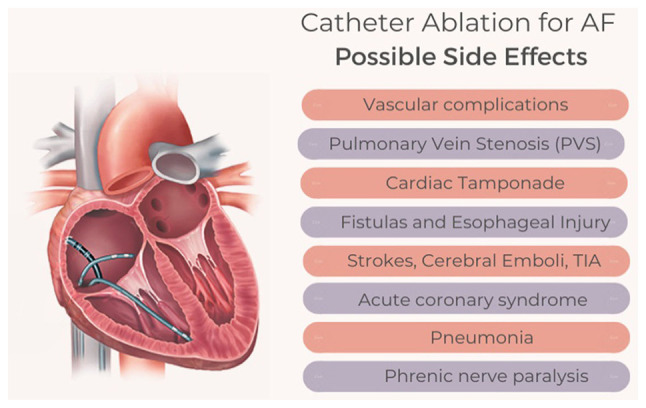
Illustration of the complications associated with catheter ablation for atrial fibrillation.

As with any medical procedure, CA for AF is not without complications. Studies have estimated that the incidence of major complications associated with CA ranges between 1 to 6.29% (86-88). In this section of the review, the specific complications associated with CA for AF that should be regularly considered by clinicians involved in its management are discussed, with the aim of effectively monitoring, preventing and managing any such complications that may arise. A summary of the complications associated with the procedure is illustrated in Fig. 1.

### Vascular complications

Vascular complications, such as access site complications, bleeding, hematoma, arteriovenous fistula and pseudoaneurysm are well-documented as the most common complications following CA. With that being said, the findings of previous studies vary when mentioning the exact rate of vascular complications (86,87,89,90). Steinbeck *et al* (89), in their study in 2018, attributed this variation to numerous reasons such as sex, an age >75 years and other comorbidities such as hypertension. Prudente *et al* (91) found that patients with an increasing age and those with AF at the time of CA were associated with a greater number of femoral vascular complications. Notably, catheter sheath size and procedure duration did not affect the complication rate (91). One interesting finding was the use of ultrasound when guiding the access to the vascular compartment and was associated with lower rates of major vascular complications (92). As expected, procedures performed in high-volume centers were associated with lower rates of complications (87).

### Pulmonary vein stenosis (PVS)

It is well-known that almost 90% of AF cases arise from pulmonary veins, which is the location where CA of arrhythmogenic foci of AF takes place. As a consequence, it was observed that weeks or months post-procedure, the narrowing of the pulmonary vein lumen can develop (93). The most commonly accepted proposed mechanism is neointimal proliferation and myocardial fibrosis (88). Generally, PVS classification depends on the degree of stenosis, where 20-50% is considered as mild, 50-69% is considered moderate, and ≥70% is considered severe (88,93). The prevalence of PVS varies in the literature. However, the majority of studies agree on a prevalence of ~40% (88,93,94) with a percentage of 0.3-3.4 for severe PVS (94). The most common presenting symptoms are shortness of breath, cough, fatigue, exercise intolerance, chest pain on exertion and hemoptysis (94). As regards diagnosis, contrast computed tomography (CT) scans and magnetic resonance imaging (MRI) remain the preferred method of choice. Perfusion scans, transesophageal echocardiograms (TEEs) and pulmonary venography are other modalities that can be utilized for diagnosis (88,94).

### Cardiac tamponade

Out of the numerous complications associated with CA for AF, cardiac tamponade remains the most common life-threatening complication (88,89). The reported incidents of cardiac tamponade range between 1-1.6% (88,89,95). On the other hand, it was previously found that an older age (>65 years), redo-procedures (95), and using two or more trans-septal punctures were associated risk factors for developing cardiac tamponade (88). Usually, the mechanism involved in this process is when puncturing occurs in the posterior right atrial wall just before entering the septum or when exiting from the left atrium towards the left atrial appendage, roof, or lateral wall (88). However, it is worth mentioning that the risk of increased bleeding into the pericardial space can be augmented, particularly when the patient is on large doses of anticoagulation medications, as is the case in numerous patients (96).

### Fistulas and esophageal injury; esophageal hematoma, atrial-esophageal fistula and atrial pericardial fistula

Esophageal perforation is an unfortunate complication that ensues during catheter manipulation in the close proximity of the posterior atrial wall and the esophagus. Although uncommon, previous research has reported that it can be detected by the use of intraprocedural TEE that is commonly utilized in most facilities during the procedures (97). Reported symptoms are dysphagia, food regurgitation and hoarseness occurring within 12 h of the the procedure. A chest CT scan and upper endoscopy can further confirm the diagnosis (87).

Similarly, atrio-esophageal and atrio-pericardial fistulas most commonly occur upon using any energy-exerting method when used against the posterior atrial wall (87). Due to its severe sequelae, atrio-esophageal fistula is known to be the most lethal complication of CA of AF (96) with a mortality rate that reaches 50%, according to the study by Sra (88). It usually presents within 2-4 weeks following CA (88) and can present with a number of symptoms, such as fever, neurological symptoms, septic shock (87), chest pain, heartburn, dysphagia, anorexia, hematemesis (96) and mortality (87), with a chest CT scan being the preferred diagnostic method (87).

### Other complications

The aforementioned complications were not the only ones mentioned in the literature. Other not uncommon complications include peri-esophageal vagal nerve injury, phrenic nerve injury, strokes, cerebral emboli, TIAs, air embolism, acute coronary artery syndrome, recurrent laryngeal nerve injury, mitral valve mechanical injury, arrhythmias, pericarditis, stiff left atrial syndrome and mortality (87,88,96,98).

## 5. Optimization of risk factors prior to the procedure

It is crucial to realize the modifiable risk factors of AF, such as hypertension, diabetes, obesity, OSA and alcohol consumption (9). Addressing these risk factors through lifestyle changes, medication, or other interventions can improve the effectiveness of CA and reduce the risk of AF recurrence (9). Moreover, modifying these risk factors can improve overall health and reduce the risk of other cardiovascular diseases, reducing morbidity and mortality. Therefore, identifying and optimizing the modifiable risk factors of AF is crucial in managing the condition and improving patient outcomes. A summary of the strategies that can be used to improve the outcomes of patients is presented in Table III.

### Hypertension

Preprocedural hypertension is one of the risk factors that have been found to be associated with an increased risk of AF recurrence following CA (99). Aggressive blood pressure (BP) control vs. standard BP control was examined in the SMAC-AF trial and the results revealed that aggressive BP control was not beneficial in reducing AF recurrence following CA (100).

In patients with resistant hypertension, renal artery denervation performed in conjunction with pulmonary vein isolation has been shown to provide better long-term AF suppression than pulmonary vein isolation alone (101).

### Diabetes mellitus (DM)

While long-term freedom from AF following CA appears to be similar in patients with and without DM (102), a higher baseline glycated hemoglobin (HbA1c) level has been linked to an increased risk of late AF recurrence in patients with DM following ablation. It is worth noting that the quality of glycemic control in the year leading up to the CA procedure for AF was significantly associated with the occurrence of AF recurrence within the 12 months following the procedure (103).

The role of pioglitazone in protecting patients with type 2 DM and paroxysmal AF from recurrent AF following CA has been investigated (104), with promising results. In patients who used pioglitazone, the success rate of a single pulmonary vein isolation procedure was significantly higher following a 2-year follow-up compared with non-users (86.3 vs. 70.7%, respectively), while the need for redo-ablation was significantly lower when compared to non-users (9.8 vs. 24.2%, respectively) (104).

### Smoking

Among patients with recurrent AF, smokers have a higher risk of arrhythmia relapse following CA compared with non-smokers. A previous study demonstrated that the 1-year AF recurrence rate following pulmonary vein isolation was significantly higher in smokers than in non-smokers (43 vs. 14%; HR, 3.19). However, the results did not reveal a significant difference in the risk of recurrence following CA between current and past smokers (105).

### Obesity

A previous meta-analysis of 23 studies identified a 27% increase in the relative risk of AF recurrence following CA in overweight or obese patients (106). Additionally, in overweight and obese patients with cardiovascular risk factors, a structured risk factor modification including weight reduction significantly improved long-term AF-free survival after ablation (87 vs. 17.8%, respectively) (107).

### OSA

Patients with OSA have a 31% higher risk of AF recurrence following CA compared to those without OSA (108). Severe OSA, defined as an apnea-hypopnea index of ≥10, was identified as an independent predictor of ablation failure (109,110).

Furthermore, as previously demonstrated, the risk of AF recurrence in patients with OSA undergoing CA increased by 57% if they did not receive concurrent continuous positive airway pressure (CPAP) therapy (108). It is noteworthy that the post-ablation recurrence rates of AF in OSA patients receiving CPAP were similar to those of patients with AF who did not have OSA (108,111).

### Alcohol consumption

Multiple studies have reported that alcohol abstinence following AF ablation can improve outcomes. Abstaining from alcohol consumption following the procedure was found to be associated with a significantly lower AF recurrence rate in comparison to patients who previously consumed alcohol or those who were currently consuming alcohol (34.1 vs. 41.9%) (112).

In another study, in obese individuals with more than one cardiometabolic risk factor, a post-ablation lifestyle intervention that included reducing alcohol intake to <30 g per week was shown to lead to improved long-term rhythm outcomes following the procedure (107).

### Dyslipidemia

Dyslipidemia has been identified as a risk factor for very late AF recurrence following CA. In a previous long-term study, hyperlipidemia was independently associated with a 4-fold higher risk of very late arrhythmia recurrence occurring more than one year after AF ablation (113).

A previous randomized study demonstrated that the short-term use of statins does not affect the outcome of AF ablation. Patients treated with 80 mg atorvastatin and those who received a placebo following the procedure had similar rates of early and late AF recurrence (114).

It was also previously demonstrated that supplementation with 1 to 4 g/day of polyunsaturated fatty acids (PUFAs) for 6 to 12 months did not significantly improve the clinical course of paroxysmal AF (115,116). However, combining 2 to 6 g/day of PUFAs with AAD therapy was associated with a significant reduction in the recurrence rate of AF following cardioversion, from 77.5 to 38.5% (117,118). Furthermore, PUFAs significantly reduced the early, but not the late recurrence rate of AF post-ablation (119).

### Effect of aggressive risk reductions in clinical trials and cohort studies

Pathak *et al* (107) conducted a cohort study to evaluate the impact of risk factors and weight management on AF ablation outcomes. Of the 281 consecutive patients undergoing AF ablation, 149 with a body mass index ≥27 kg/m^2^ and ≥1 cardiac risk factor were offered risk factor management (RFM) according to the AHA/ACC guidelines (107). The lifestyle treatment program included special assistance with weight management, an exercise prescription of 200 min of moderate exercise per week, advice regarding salt restriction, lipid management, glucose monitoring and treatment, smoking and alcohol counseling, and evaluation for sleep apnea (107). There were no differences in baseline characteristics, the number of procedures, or follow-up duration between the groups (P-value, not significant). RFM resulted in greater reductions in weight (P=0.002) and blood pressure (P=0.006), and improved glycemic control (P=0.001) and lipid profiles (P=0.01) (107). At follow-up, AF frequency, duration, symptoms and symptom severity decreased to a greater extent in the RFM group compared with the control group (all P<0.001) (107). Single-procedure drug-unassisted arrhythmia-free survival was greater in patients offered RFM compared with the control subjects (P<0.001) (107). Multiple-procedure arrhythmia-free survival was markedly better in patients offered RFM compared with the control subjects (P<0.001), at 16 and 42.4%, respectively, using AADs (P=0.004) (107). Overall, aggressive RFM improved the long-term success of AF ablation (107).

### Utilization of imaging for improving the outcomes of patients undergoing CA

The mapping and preparation for AF ablation is crucial to ensure successful treatment outcomes. In recent years, advances in imaging techniques, such as ultrasound and MRI, have played a crucial role in identifying arrhythmogenic substrates prior to electrophysiology studies (120,121).

Ultrasound imaging, also known as echocardiography, is a non-invasive tool that utilizes high-frequency sound waves to produce images of the heart. The use of ultrasound in AF ablation procedures has increased in recent years, with the development of three-dimensional (3D) imaging techniques. 3D ultrasound can provide a comprehensive view of the heart, including the atria and surrounding structures, allowing for more precise mapping of the arrhythmogenic substrates (120,122).

MRI is another non-invasive imaging tool that uses a magnetic field and radio waves to produce images of the heart and surrounding structures. MRI can provide detailed images of the heart's anatomy, including the location and extent of fibrous tissue and scarring, which can contribute to AF. This information can be useful in planning AF ablation procedures and in determining the appropriate ablation strategy (120,121).

Gimelli *et al* (121) conducted a study to evaluate the use of multi-modality imaging in the identification of arrhythmogenic substrates prior to electrophysiology studies. Their study found that the combination of ultrasound and MRI provided a comprehensive understanding of the underlying causes of AF, including the location and extent of fibrous tissue and scarring. This information allowed for a more precise and effective ablation strategy.

In conclusion, the use of imaging techniques, such as ultrasound and MRI, has greatly improved the mapping and preparation for AF ablation procedures. These tools provide critical information on the arrhythmogenic substrates, allowing for more precise and effective ablation strategies. The findings from the study by Gimelli *et al* (121) demonstrate the importance of multi-modality imaging in the identification of arrhythmogenic substrates prior to electrophysiology studies.

## 6. Use of antiarrhythmic and anticoagulation agents following the procedure

### Use of AADs

Conflicting data exist regarding the optimal strategy for AAD therapy following CA in patients with AF (9,25). A previous multicenter, registry-based prospective study was performed in Germany on 3,275 patients undergoing CA (123). That study compared the outcomes of patients who were started on AADs vs. those who were not, at the time of discharge. The results were further analyzed in subgroups of patients with paroxysmal AF and those with persistent AF (123). That study found that at 12 months following the procedure, patients who were discharged and treated with AADs exhibited similar rates of recurrence, rehospitalization and cardiovascular events compared to those who were not on AADs. However, in the subgroup of patients with paroxysmal AF, the use of AAD at discharge was linked to lower treatment satisfaction and a higher rate of repeat ablation (123). The lower treatment satisfaction was concluded by the patients rating the procedure as ‘non-successful’ more often when they are discharged on AADs. This finding can be explained by the desire of the patients to terminate antiarrhythmic medications to consider the procedure successful.

On the other hand, a multicenter RCT was conducted on a total 153 patients with paroxysmal AF who underwent pulmonary vein isolation (124). That study included only 153 patients who had continued taking the previously ineffective AADs during a 3-month ‘blanking period’. Patients who developed recurrence during this period were excluded from the study. At the end of the blanking period, patients who remained free of AF were randomly assigned to one of two groups: A group in which AADs were continued after the procedure and another group in which AADs were discontinued (124). The results revealed that continuing the use of AADs significantly reduced the recurrence of atrial tachyarrhythmias in the first year after pulmonary vein isolation (124). However, it should be noted that patients who had a recurrence during the blanking period were not studied. By the nature of the study protocol, only patients who were continued on AADs in the first 3 months were recruited, which may have increased their dependence on AADs increasing the possibility of recurrence after discontinuation. Additionally, that study was not blinded which may have been a source of bias.

### Use of anticoagulants

The safety of discontinuing oral anticoagulant (OAC) therapy following AF ablation remains controversial. Current practical clinical guidelines (9,25) recommend continuing OAC therapy for stroke/thromboembolism for at least 2 months in all patients, regardless of stroke risk factors. Beyond this time, the decision to continue or terminate OAT should not be based on the apparent success or failure of CA for AF or the pattern of AF, but on the stroke (CHA2DS2-VASc score) and bleeding risks (HAS-BLED score) and comorbidities of the patient (9,25). However, the estimated bleeding risk, in the absence of absolute contraindications to OAC therapy, is not recommended to guide the decision to use OAC therapy for stroke prevention. Patients who are at a high risk of stroke (i.e., CHA2DS2-VASc score ≥2 for males or ≥3 for females, prior history of stroke), in whom the reduction in the risk of a disabling stroke may outweigh the risk of bleeding. For patients with an intermediate risk of stroke (CHA2DS2-VASc score 1 in males or 2 in females), long-term OAC therapy is also recommended. Finally, in patients who are at a low risk of stroke (CHA2DS2-VASc score of 0 in males or 1 in females), the risk of stroke/systemic embolism in observational studies is very low (0-0.2%), and the risk of bleeding associated with long-term OAC therapy outweighs the benefits of stroke prevention; thus, the discontinuation of OAC therapy should be considered 2 months post-CA for AF, regardless of AF recurrence (9,25).

The current evidence evaluating the safety of discontinuing anticoagulation in patients with AF following successful CA is limited to observational studies. The large observational study conducted by Karasoy *et al* (125) reported the outcomes of 4,050 patients with AF undergoing first-time radiofrequency CA. Among the 1,507 patients with n increased risk of stroke (CHA_2_DS_2_VASc score ≥2), OAC was discontinued in 30% of patients at 1 year. The overall rate of thromboembolism was low and comparable between patients with discontinued OAC (0.93 per 100 patient-years) and continued OAC use (0.97 per 100 patient-years).

Proietti *et al* (126) conducted a systematic review of 10 prospective cohort and 6 retrospective cohort studies (25,177 patients) that reported cerebrovascular events (CVE) following CA for AF and compared patients treated with OACs vs. those who were not treated with OACs. There were no significant differences in the incidence of CVE between patients treated or not with OACs following CA for AF. Patients not treated with OACs suffered significantly less bleeding than those on-OAT with a relative risk of 0.17 (CI, 0.09-0.34); however, they had lower CHADS2 scores than those treated with OACs, probably reflecting the reluctance of clinicians to discontinue OAC therapy in patients who were at a high thromboembolic risk and the influence of current guidelines.

Additionally, a recent meta-analysis of prospective studies was conducted by Liu *et al* (127) to assess the safety and feasibility of discontinuing OAC therapy following successful AF ablation. Their study included 11,148 patients (7,160 in the off-OAC group and 3,988 in the on-OAC group) where no significant difference in thromboembolism between both groups was observed with an odds ratio of 0.73 (95% CI, 0.51-1.05; I^2^=0.0%) (127). In addition, their study concluded that the risk of major bleeding in the off-OAC group was significantly lower compared to the on-OAC group with an odds ratio of 0.18 (95% CI, 0.07-0.51; I^2^=51.7%). Overall, that study provides evidence suggesting that it may be safe to discontinue OAC therapy in patients following successful AF ablation. Additionally, an increased risk of major bleeding was observed in patients on OACs (127).

Regrettably, there is still a lack of prospective randomized clinical trials to recommend whether it is safe or not to discontinue OAC therapy following successful CA for AF in patients with intermediate-to-high stroke risk and the decision of whether OAC can be safely discontinued post-CA for AF remains controversial. Currently, continued long-term OAC guided by the stroke risk factor profile is the only proven strategy to prevent stroke. A summary of the recommendations regarding the use of anticoagulant and antiarrhythmic agents following the procedure is presented in Table IV.

## 7. Cost-effectiveness of the procedure

The determination of cost-effectiveness relies heavily on deduced results from clinical trials that are characterized by limited durations of follow-up and inadequate sample sizes, necessitating the formulation of conjectures regarding critical clinical outcomes (128,129). In order to enhance the effectiveness of these estimations, it is imperative to secure comprehensive data from more extensive and prolonged studies. This will provide a more nuanced and informed perception of the long-term costs and benefits associated with treatment enabling the refinement of cost-effectiveness calculations.

Furthermore, the costs associated with AF ablation procedures can fluctuate significantly based on the settings of treatment and the type of equipment utilized (130,131). Estimations of the cost-effectiveness of AF ablation can be further affected by a multitude of factors, such as the patient demographics, the magnitude of symptoms, the duration of analysis, and the assumptions made regarding the effect of AF ablation on QOL, strokes and other clinical outcomes.

The argument of the cost-effectiveness of AF ablation is based on the premise that over time, the expenses incurred from the procedure can be partially compensated by the reduction of healthcare resource utilization that stems from the long-term management of arrhythmias in patients who do not receive ablation. This notion is supported by some empirical evidence (132-134). However, the majority of formal cost-effectiveness studies have not been able to establish that AF ablation is cost-neutral or cost-saving in the short to intermediate term. This highlights the need for longer-term studies when considering the cost-effectiveness of AF ablation.

The cost-effectiveness of AF ablation has largely been evaluated in comparison to AAD therapy as a second-line treatment option for patients with paroxysmal AF (131,135-138). These studies have generally produced favorable cost-effectiveness ratios (136,138). However, the cost-effectiveness of AF ablation as a first-line therapy, particularly in patients with persistent or long-term persistent AF, is not as well established. On the other hand, one study based on the MANTRA-PAF trial population suggests that AF ablation may only be cost-effective as a first-line treatment in younger patients (139).

## 8. Conclusion

CA holds promise as an effective therapeutic strategy for the management of AF. However, careful patient selection, procedural expertise and the consideration of individual patient characteristics are crucial for optimizing outcomes. Further research is required in order to clarify the role of CA in specific patient populations and its impact on stroke prevention.

## Figures and Tables

**Table I:** Summary of indications and recommendations for catheter ablation in atrial fibrillation (9,25).

Indication	Level of evidence	Recommendations
Catheter ablation as a first-line therapeutic option	IIa	Symptomatic paroxysmal AF: Reasonable as a primary treatment option without a trial of antiarrhythmic agents.
	Ia	In selected patients with symptomatic paroxysmal AF: Consider as a first- line treatment option, particularly in younger patients with few comor- bidities and where rhythm control is desired.
	IIa	Symptomatic persistent AF: Reasonable as a primary treatment option without a trial of antiarrhythmic agents.
	IIb	Long-standing symptomatic AF: Can be considered as a primary treatment option without a trial of antiarrhythmic agents.
	IIa	Symptomatic pauses (tachy-brady syndrome): Reasonable as a primary treatment option. Highly competitive athletes: Recommended as a primary treatment option.
Catheter ablation as second- line treatment	Ia	Recommended for patients who have failed one or more antiarrhythmic medications as a second-line treatment option.
Catheter ablation in patients with heart failure and reduced ejection fraction (HFrEF)	IIb	Can be considered as a treatment option for patients with heart failure with reduced ejection fraction. Reasonable indications for patients with heart failure: AF ablation in selected patients with heart failure is reasonable, following similar indications to those without heart failure. Associated with a lower risk of all-cause mortality and improved left ventricular ejection fraction (LVEF) compared to medical therapy.
Catheter ablation in patients with heart failure and pre- served ejection fraction (HFpEF)	IIb	Can be considered as a treatment option for patients with HFpEF. Similar indications as for patients without heart failure: AF ablation is reasonable in selected patients with HFpEF. Effective in maintaining sinus rhythm, reducing heart failure rehospita- lizations, and improving quality of life.
Catheter ablation in elderly patients	IIa	Reasonable to consider for selected older individuals with AF, following similar indications to younger patients. Higher complication rates, including cerebrovascular accidents, bleeding, and mortality, compared to younger patients.
Catheter ablation in asymp- tomatic patients	IIb	May be considered for selected patients with persistent or paroxysmal AF; however, the benefit in asymptomatic patients remains uncertain, and further research is required.
Catheter ablation to reduce stroke risk		Clinical trials and meta-analyses do not support a significant reduction in stroke risk compared to medical therapy.

AF, atrial fibrillation.

**Table II:** Summary of the outcomes following catheter ablation for atrial fibrillation.

Outcome	Success rate (no recurrence)	Quality of life	Mortality
Single procedure	65% at 1 year, 56% at 3 years, 51% at 5 years	Improved in both symptomatic and asymptomatic patients	No significant difference compared to medical therapy
Multiple procedures	86% at 12 months, 79% at 3 years, 78% at 5 years	Sustained improvements in quality of life scores	Significantly lower in patients with stable sinus rhythm and no recurrence^a^

^a^Observation from cohort studies and not from clinical trials (63).

**Table III:** Strategies used to improve the outcomes of patients undergoing CA for AF.

Risk factor	Modification	Effect on AF ablation outcomes
Hypertension	Aggressive blood pressure control	Improved long-term AF suppression
Diabetes mellitus	Improved glycemic control	Improved long-term AF suppression
Smoking	Smoking cessation	Improved long-term AF suppression
Obesity	Weight loss	Improved long-term AF suppression
Obstructive sleep apnea	Continuous positive airway pressure (CPAP) therapy	Improved long-term AF suppression
Alcohol	Alcohol abstinence	Improved long-term AF suppression
Dyslipidemia	Statin therapy	May improve long-term AF suppression
Polyunsaturated fatty acids (PUFAs)	Supplementation	May improve early, but not late AF recurrence rate
Aggressive risk reductions	Lifestyle modification and risk factor management	Improved long-term AF suppression
Imaging	Ultrasound and MRI	Improved long-term AF suppression

CA, catheter ablation; AF, atrial fibrillation.

**Table IV:** Summary of the recommendations regarding the use of anticoagulant and antiarrhythmic agents following the procedure.

Factor	Recommendation	Evidence	Risks	Benefits
Antiarrhythmic drugs	Continue or discontinue based on individual patient factors	Conflicting data	-	May reduce the risk of the recurrence of atrial fibrillation
Anticoagulation therapy	Continue for at least 2 months, then consider discontinuation based on individual patient factors	Conflicting data	Bleeding	May reduce the risk of stroke

## Data Availability

Not applicable.
